# Genetic influences on the association between DNA methylation and obesity measures: insights from a twin study design

**DOI:** 10.1186/s13578-025-01446-2

**Published:** 2025-07-23

**Authors:** Xuanming Hong, Hui Cao, Weihua Cao, Jun Lv, Canqing Yu, Tao Huang, Dianjianyi Sun, Chunxiao Liao, Yuanjie Pang, Runhua Hu, Ruqin Gao, Min Yu, Jinyi Zhou, Xianping Wu, Yu Liu, Shengli Yin, Wenjing Gao, Liming Li

**Affiliations:** 1 Department of Epidemiology and Biostatistics, School of Public Health, Peking University, Beijing, 100191 China; 2 Key Laboratory of Epidemiology of Major Diseases, Ministry of Education, Peking University, Beijing, 100191 China; 3 Qingdao Center for Disease Control and Prevention, Qingdao, 266033 China; 4 Zhejiang Center for Disease Control and Prevention, Hangzhou, 310051 China; 5 Jiangsu Center for Disease Control and Prevention, Nanjing, 210008 China; 6 Sichuan Center for Disease Control and Prevention, Chengdu, 610041 China; 7 Heilongjiang Center for Disease Control and Prevention, Harbin, 150090 China; 8 Dezhou Center for Disease Control and Prevention, Dezhou, 253016 China

**Keywords:** DNA methylation, Obesity, Genetic correlations, Twin study

## Abstract

**Background:**

Both obesity and DNA methylation (DNAm) are influenced by genetic factors. Despite more than a thousand of obesity-related DNAm sites (CpGs) being identified, studies that account for genetic influences in these associations are limited.

**Results:**

Using data from 1,074 twins in the Chinese National Twin Registry and bivariate structural equation models (SEMs), we investigated the phenotypic (Rph), genetic (Ra), and environmental (Re) correlations between genome-wide DNAm and three obesity indices: BMI, waist circumference (WC), and waist-to-hip ratio (WHR). Genome-wide, correlations between DNAm and obesity were small (Rph = 0.04, Ra = 0.08–0.09, Re = 0.02–0.03). For CpGs with high phenotypic correlation (Rph > 0.1), the mean genetic and environmental correlations were 0.23–0.24 and 0.03–0.05, respectively, indicating significant genetic influence on the DNAm-obesity associations. To further investigate the role of genetic influences, we then categorized the CpGs into different groups: high phenotypic correlation (Rph ≥ 0.2); high phenotypic and genetic correlations (Rph > 0.1 and Ra > 0.5); high phenotypic and low genetic correlations (Rph > 0.1 and Ra < 0.5). Association studies were conducted in the full population and in the monozygotic (MZ) twin-paired design, where genetic influences were controlled. For CpGs with Rph ≥ 0.2, 9, 8, and 22 were associated with BMI, WC, and WHR in the full population, but only 6, 1, and 1 CpGs remained significant after controlling for genetic effects in MZ twin-pair analyses. For CpGs with Rph > 0.1 and Ra > 0.5, genetic factors predominantly drove the association, and none of the 155/155/189 CpGs associated with BMI/WC/WHR in the full population were significant in MZ-paired analyses. For CpGs with Rph > 0.1 and Ra < 0.1, genetic effects were minimal or confounding, with 89, 4, and 17 significant in both full population and MZ-paired analyses.

**Conclusions:**

Our results highlight the significant genetic influences on the DNAm-obesity relationships, which may explain the low replicability of obesity-related DNAm markers. This indicates that genetic influences should be carefully considered in DNAm-related studies.

**Supplementary Information:**

The online version contains supplementary material available at 10.1186/s13578-025-01446-2.

## Introduction

Globally, obesity and related comorbidities impose a major burden on public health, economy, and mental well-being [1]. Therefore, understanding the mechanisms influencing the obesity is a crucial health priority. The pathophysiology of obesity is multifactorial, influenced by complex interactions between genetic, epigenetic, and environmental factors [2]. In recent years, extensive efforts to identify genetic determinants of obesity indicators through genome-wide association studies (GWAS) have identified over thousands of single nucleotide polymorphisms (SNPs) associated with related measures. However, in one of the most extensive GWAS conducted for BMI to date, 941 significant lead SNP account for only 6% of the variation in BMI, individually of small effect size [3].

On the other hand, DNA methylation (DNAm) may help capture a greater proportion of interindividual variation in BMI [4]. As a stable epigenetic changes, DNAm can reflect a wide range of genetic influences and environmental exposures at the molecular level [4, 5]. By altering gene expression and stabilizing chromatin structure, DNAm has been implicated in the susceptibility of multiple diseases including obesity [6]. The relationship between obesity and methylation has been explored in numerous studies at various scales, more than one thousand genes and associated 5′-cytosine-phosphate-guanine-3′sites (CpGs) have been identified as being related to the obesity-related measures [7, 8]. Nevertheless, the reproducibility of significantly associated CpG sites across diverse studies remains inadequate. Genetic variations among different study populations may primarily account for the observed inconsistencies in these results. Genetic factors, such as methylation quantitative trait loci (meQTLs), have been shown to significantly influence the interpretation of these findings and therefore must be considered in DNAm studies [9, 10]. Numerous researchers have emphasized the importance of accounting for genetic factors in methylation-related studies [11]. Typically, CpG sites with SNPs within 10 base pairs and a minor allele frequency (MAF) greater than 0.01 are excluded in most DNA methylation studies to minimize the genetic influences [12]. However, there is a paucity of studies that specifically explore the influence of genetic factors on the association between DNAm and phenotypes. In light of these considerations, for studies investigating the associations between DNAm and obesity, a key scientific question is whether these associations are independent of genetic factors, or if they are primarily driven by genetic factors simultaneously influencing both DNA methylation and obesity.

Twin studies provide a valuable research tool for exploring the relationship among genetic factors, DNAm, and obesity indicators. The genetic or environmental factors underlying phenotypes can be controlled as confounding variables to some extent in twin studies [13]. Based on the shared genetic background of twins, by utilizing the phenotype of the co-twin as an instrumental variable for the twin individual, researchers have investigated the potential causal relationships between BMI and DNAm at specific CpGs [14]. Additionally, based on the degree of shared genetic background between monozygotic and dizygotic twins, phenotypic variation can be evaluated to estimate the extent of genetic influence. Several research teams have applied bivariate structural equation modeling (SEMs) in twin populations to quantitatively estimate the shared genetic and environmental factors between specific CpGs and phenotypes such as blood pressure and metabolic syndrome [15, 16].

Based on data from the Chinese National Twin Registry (CNTR), this research aims to ① investigate the genome-wide genetic and environmental correlations between DNAm and obesity measures, and ② identify the associations between DNAm and obesity indicators under both uncontrolled and genetically controlled conditions. Understanding the genetic contributions underlying the associations between DNAm and obesity measures, particularly through the comparison of results from two different association studies, enhances our comprehension of obesity-related genetic and epigenetic changes.

## Methods

### Study population

The data utilized in this study were derived from the CNTR. Comprehensive details of the CNTR’s study design, data collection process, and participant characteristics have been previously described [17]. The CNTR thematic survey data collection was conducted twice in 2013 and 2018, respectively. The initial CNTR special survey cohort enrolled a total of 3,038 twin individuals, from whom data were collected through standard questionnaires, physical examinations, and blood samples. The questionnaires include various topics such as demographic information, medical history, and lifestyle habits. Physical examinations measured BMI, waist circumference (WC), hip circumference (HC), height, and weight. DNAm assays and biochemical analyses were conducted on whole blood samples. The study protocol received ethical approval from the Biomedical Ethics Committee at Peking University, Beijing, China, and all participants provided written informed consent (reference numbers: IRB00001052-22032, IRB00001052-13022, IRB00001052-14021).

Inclusion criteria for this study included: (1) completion of questionnaires and physical examinations for both twins; (2) availability and completion of blood withdrawal from both twins; (3) participation of twin pairs in at least one detailed survey conducted in either 2013 or 2018. Exclusion criteria involved pregnant females and their cotwins. Following the application of inclusion and exclusion criteria, the initial twin cohort for this study was selected through a systematic random sampling strategy for DNAm examination, consisted of 1,088 participants (mean age = 49.9 ± 12.2) [17]. If a twin was excluded during subsequent DNAm data quality control or statistical analysis, the cotwin was also excluded.

### Measurement of body mass index, waist circumference, waist-to-hip ratio, twin zygosity and covariates

The height, weight, WC, and HC of each sample were measured twice repeatedly during both the 2013 and 2018 surveys. If the difference between the initial two measurements exceeded 1 cm for height, WC, or HC, a third measurement was performed. The mean values derived from the two closest measurements were used for subsequent analyses. Weight was assessed using a MC-780 body composition monitor. WC was assessed at the midpoint between the lower rib and the iliac crest with a tape measure. HC was assessed at the broadest circumference below the waist. BMI was computed by dividing the mean weight (kg) by the square of the height (m [2]), while the WHR was calculated by dividing WC by HC.

Zygosity determination for twins was conducted using a panel of 59 SNPs from the Illumina Infinium Methylation Chips. Twins with over 90% identical SNPs were classified as monozygotic (MZ) twins [18]. In this study, 758 twins (379 pairs) were initially identified as MZ, whereas 330 twins (165 pairs) were classified as DZ.

Smoking status was categorized into three groups: current, former, and never smokers [19]. Analogously, drinking status was classified into current, former, and never drinkers [20].

### DNA methylation assessment

DNA samples were extracted from peripheral blood and modified to bisulfite using the EZ DNA Methylation Kit (Zymo Research, Orange County, CA, USA). Genome-wide DNA methylation profiles were quantified using the Illumina Infinium Human Methylation 450 K or EPIC BeadChips (Illumina, San Diego, CA, USA). Probes presented in both platforms were included in the subsequent analyses. An inter-assay reproducibility has been investigated to be 98% between the two BeadChips. It was observed that 90% of the probes on the 450 K microarray were replicable with the EPIC BeadChip [21]. Data from the EPIC and 450 K platforms were combined using the “combineArrays” function in the R package “minfi” (version 1.34.0) to create a combined dataset [22].

Methylation levels for each CpG site were presented as β-values, representing the average methylation level on a scale from 0 (fully unmethylated) to 1 (fully methylated). Quantile normalizations and adjustments for blood cell proportions were then conducted for these β-values were using the “ChAMP” package (version 2.18.3), with quality control measures applied to filter out low-quality detection probes and samples. To address batch effects, the ComBat method was applied to the DNAm data after quality control using the “sva” package. The detailed information on these processes is provided in the supplementary materials.

In total, 378,654 CpGs that were present on both the 450k and EPIC arrays, and 1,074 participants with DNAm data (comprising 756 MZ twins [378 MZ pairs] and 318 DZ twins [159 DZ pairs]), were included in the subsequent analysis.

### Retrieval of SNP loci corresponding to CpGs and obesity indicators

SNP loci corresponding to CpGs (meQTL) were derived from a recent study, which validated the relationship between genetic variation and DNA methylation based on blood samples from 3,799 Europeans and 3,195 Asians. The study finally identified 11,165,559 high-confidence and highly reproducible SNP–CpG associations (meQTLs), including 2,709,428 SNPs and 70,709 CpG sites (Supplementary Materials). These meQTLs comprised both cis and trans types: 10,346,172 cis meQTLs (SNP-CpG distance < 1 Mb) and 467,915 trans meQTLs (SNP-CpG distance > 1 Mb or on different chromosomes). The study employed stringent statistical thresholds (*P* < 10^− 14^) to define meQTLs and utilized fastenloc for co-localization analysis of trans meQTLs with GWAS data. Additionally, Summary-based Mendelian Randomization (SMR) analysis was used to assess the covariation between proximal candidate genes and trans methylation signatures. Covariates included fixed-effect meta-analysis and multiple testing correction using the Benjamini-Hochberg method [23].

SNP loci associated with indicators of obesity was obtained by systematically searching for genome-wide association studies on BMI/WC/WHR. Online databases PubMed and EMBASE were systematic searched. The search for published studies was limited up until November 18, 2024. Details on the search strategy and selection criteria are provided in the supplementary materials.

After obtaining the SNP loci, we paired the corresponding obesity measures and CpG sites associated with the same SNP loci for a subsequent analysis.

### Statistical analysis

The statistical analyses were performed in three major steps using R (R statistics), version 4.3.1.

### Genetic correlations analysis

For genome-wide CpG sites, we conducted bivariate structural equation models (SEMs) to test the extent to which the correlations between the DNAm levels and obesity measures were driven by genetic contributions in the complete analysis cohort of 1,074 participants (including 756 MZ twins and 318 DZ twins). Bivariate SEMs can be used to estimate genetic and environmental contributions that are shared between different two variables, as well as those that are uniquely attributed to each variable [24]. The intricacies of Bivariate SEMs have been previously detailed (see Supplementary Fig. 1) [25]. Briefly, the phenotypic variation of DNAm and the obesity indicators were partitioned into ①additive genetic variance (A), representing the cumulative effect of alleles; ②nonadditive genetic variance (D), representing the effects resulting from interactions between alleles; ③common environmental variance (C), arising from environmental effects shared by twin pairs; ④and unique environmental variance (E), attributable to environmental effects that are not shared between twin pairs.

To recognize the genetic and environmental contributions to the observed covariance between DNAm and obesity indicators, a sequence of submodels were employed. These submodels evaluated whether the genetic, shared environmental, and unique environmental paths from DNA methylation levels to obesity measures could be constrained to 0. Given that the C and D components cannot be differentiated in families where twins are raised together, ACE (models including additive genetic[A], common [C] and unique environmental variance [E]) and ADE (models including additive genetic[A], nonadditive genetic variance [D], and unique environmental variance [E]) models were fitted separately for each site, with the optimal model selected based on the lowest AIC value. For example, in the ACE model, if the shared genetic path (a21) from DNAm levels to obesity measures could not be constrained to 0, an overlap between the genetic factors influencing both DNAm and obesity measures were indicated. Genetic and environmental correlations (Ra and Re) between the traits were calculated from the variance/covariance matrix of the bivariate SEM, illustrating the shared components between these phenotypes, which can vary between 0 and 1. The Ra and Re can also be interpreted as the extent to which genetic and environmental factors contribute to phenotypic correlations (Rph). In this study, we consider a Ra > 0.5 to indicate high genetic correlation.

The analyses were adjusted for age and sex. One-sided *P*-values < 0.05 were considered as statistically significant.

### Identification of the associations between CpG sites and obesity measures

The associations between CpG sites and obesity indicators were assessed by establishing linear mixed-effect (LME) models. In the complete analysis cohort of 1,074 participants, the DNAm levels at each CpG site were considered as the continuous dependent variables, with obesity indicators as independent variables. Covariates including age, sex, smoking status, alcohol consumption were included as fixed effects in the LME models. To account for within-pair effects, twin ID and zygosity (MZ or DZ) were incorporated as random intercept terms.

To assess whether the genetic effects influence the associations between CpGs and obesity measures, a paired-twin analysis was conducted exclusively on MZ twins (within-pair analysis, *n* = 756). This method further controlled for genetic background underlying the relationships. In this analysis, LME models were also employed to examine the associations between differences in BMI, WC, and WHR, and DNA methylation levels within MZ twin pairs. The within-pair difference was calculated by subtracting the trait value of one twin from that of their co-twin. Fixed effects included smoking status and alcohol consumption, while twin ID was used as a random effect term to account for the dependency between twins within a pair. The formula of the LME model is as follows: Y = X_diff_+ X_mean_+Covariate_self_+TID(random effect term), the Y indicates the values of dependent variables, X_diff_ indicate the differences in the values of independent variables, obtained by subtracting those of their co-twin from that of each individual twin, while X_mean_ denotes the mean values of independent variables within twin pairs [26].

Benjamini–Hochberg (BH) adjustment was used to control the false discovery rate (FDR) in multiple testing and identify significant results (*P* < 0.05) in the association study.

**Fig. 1:**
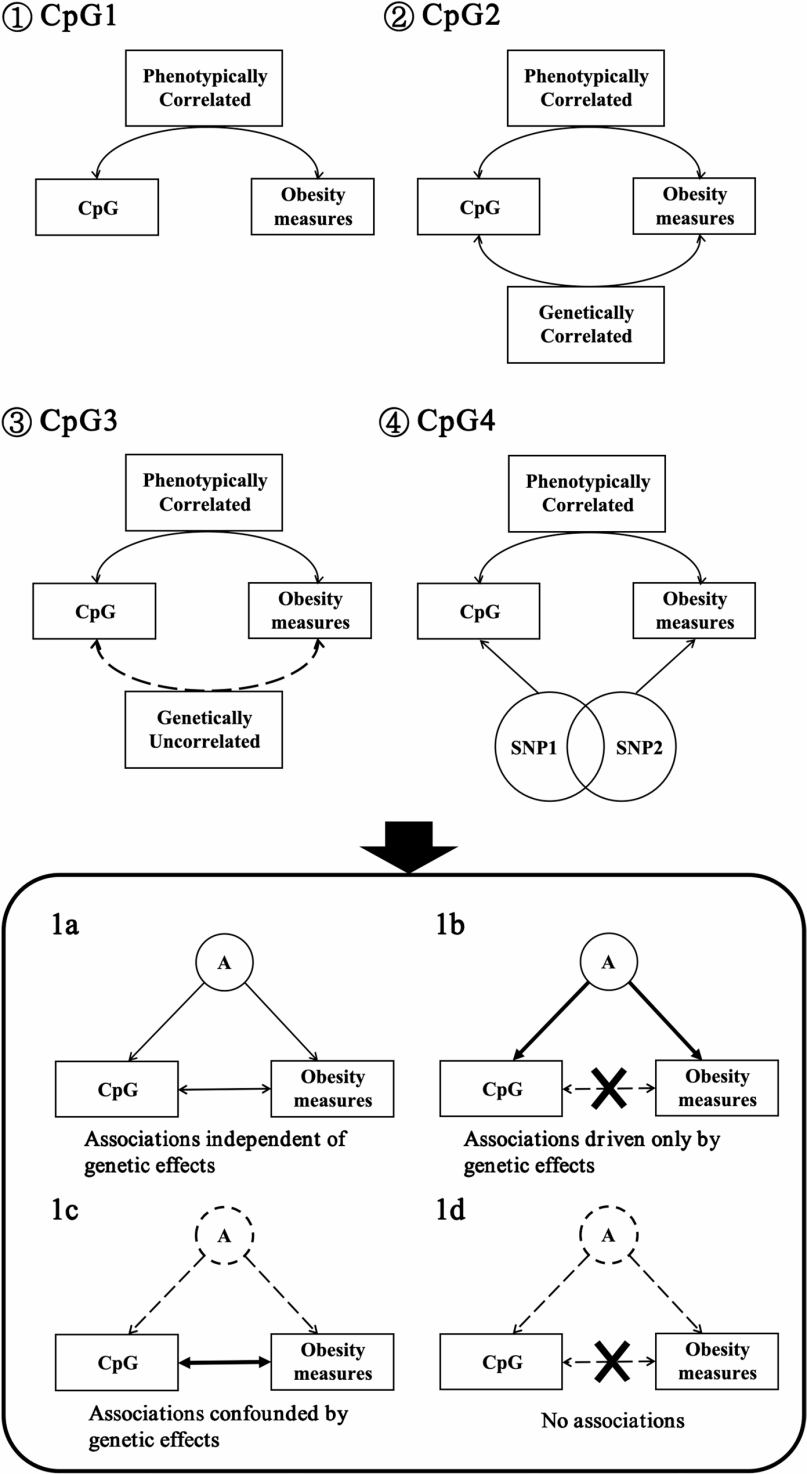
Four Potential Outcomes from Association Analyses. ①②③④indicate the four distinct groups of CpG sites that categorized prior to conducting association analyses described as above

To facilitate the evaluation of the influence of genetic factors on the association between DNAm and obesity indicators, we conducted general association analyses and MZ-paired association analyses in four distinct groups of CpG sites, defined as follows, as well as in Fig. 1:

①CpG sites with significant Rph > = 0.2.

②CpG sites with significant Rph > 0.1 and significant Ra > 0.5 for obesity-related indicators.

③CpG sites with significant Rph > 0.1 for obesity-related indicators, but showing nonsignificant Ra with point estimates < 0.1.

④CpG sites with significant Rph > 0.1 and sharing common SNPs with the obesity-related indicators, suggesting a potential shared genetic basis.

To illustrated, we used the BMI-associated SNPs and meQTLs obtained from previous studies, and extracted the intersect of these SNPs to define CpGs in group ④.

Figure 1a, b, c, and d illustrate the four potential results from association analyses. (1a) The association between CpG and obesity measure is independent of genetic effects. (1b) Genetic effects primarily drive the association between CpG and obesity measure. (1c) Genetic effects confound the association between the CpG site and the obesity measure. (1d) The CpG site is neither associated with nor genetically correlated with the obesity measure.

The “SNPs1” and “SNPs2” within the circles refer to SNPs associated with CpGs and obesity phenotypes, respectively, with some overlap between them. “A” denotes the genetic contributions.

**Fig. 2:**
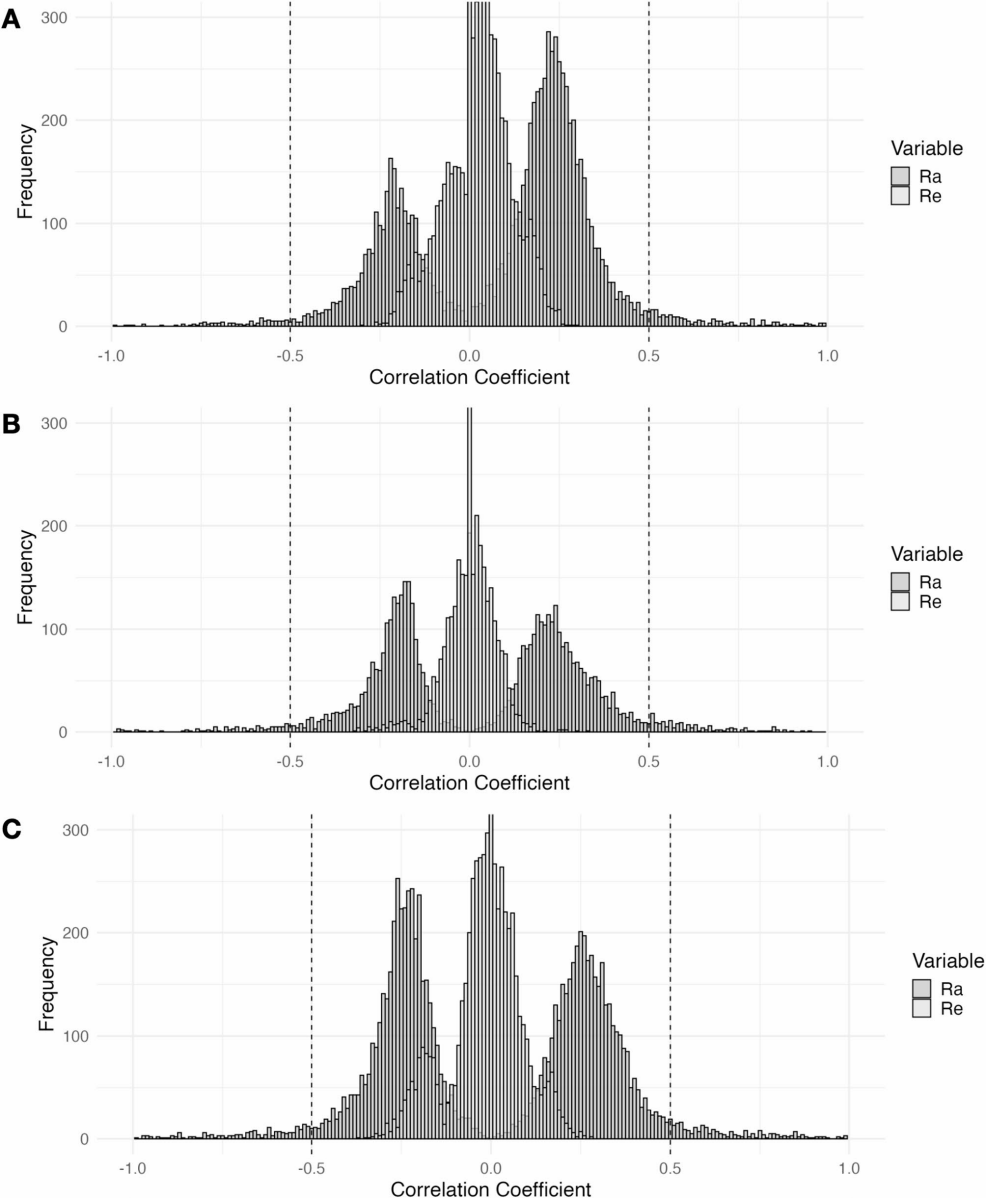
Genetic and Environmental Correlations Between DNA Methylation and Obesity-Related Traits for CpG Sites with Phenotypic Correlations Exceeding 0.1. (**A**), (**B**), and (**C**): Histograms of bivariate structural equation modeling estimates for the correlations between DNAm and BMI, WC, WHR, Respectively. Ra, genetic correlation; Re, environmental correlation

Four potential outcomes can be yielded from the association analyses (Fig. 1a-d). First, if the association is significant in both the full population and the MZ-paired analysis (i.e., Fig. 2a), it indicates that the association is independent of genetic effects. Second, if the association between CpG and obesity measures is significant in the full population analysis but not in the MZ-paired analysis (Fig. 1b), it suggests that genetic effects predominantly driven this association. Third, if the association is not significant in the full population analysis but is significant in the MZ-paired analysis (Fig. 2c), it implies that genetic effects may confound the association between CpGs and obesity indicators. Lastly, a CpG site may neither be associated with the corresponding obesity measure nor genetically correlated with it. This possibility is observed in CpGs from the ④ group.

The annotation file for the Illumina Infinium EPIC array was employed to annotate the CpGs.

Following the association analyses, pathway enrichment analyses were performed for the significant CpGs based on ①-④ group, utilizing the “enrichGO” function from the “clusterProfiler” package. Human gene annotation was conducted using the “org.Hs.eg.db” package. Pathways that demonstrated an FDR adjusted *p*-value < 0.05 were recognized as having statistical significance.

## Results

### Characteristics of the study population

Table 1 describes the detailed demographic information of the participants. The 1,074 participants had a mean age of 49.9 (SD = 12.2) years old and included 756 MZ twins. The mean BMI was 24.8 kg/m^2^ (SD = 3.9), with a mean WC and WHR of 86.9 cm (SD = 10.8) and 0.9% (SD = 0.1%), respectively. For males, the mean BMI was 25.2 (SD = 4.0), mean WC was 88.7 cm (SD = 10.0), and mean WHR was 0.9 (SD = 0.1). For females, the mean BMI was 24.1 (SD = 3.6), mean WC was 83.0 cm (SD = 11.4), and mean WHR was 0.9 (SD = 0.1). The overall within-pair correlations for BMI, WC, and WHR in the entire cohort of twins were 0.4, 0.4, and 0.3 (all *P* < 0.01), respectively. When analyzed by zygosity, MZ twins exhibited correlations of 0.6 for BMI, 0.6 for WC, and 0.4 for WHR (all *P* < 0.01), whereas DZ twins showed correlations of 0.1 (*P* = 0.68), 0.2 (*P* = 0.04), and 0.2 (*P* = 0.01) for BMI, WC, and WHR, respectively.

**Table 1:** Characteristics of the analytic samples by study group

		Total
N		1,074
Age, yrs		49.9 ± 12.2
Female, n (%)		341 (31.8)
MZ, n (%)		756 (70.4)
BMI, kg/m^2^		24.8 ± 3.9
WC (cm)		86.9 ± 10.8
WHR (%)		0.9 ± 0.1
Smoking status, n(%)		
	Current smoker	353(32.9)
	Former smoker	140(13.0)
	Nonsmoker	581(54.1)
Alcohol consumption, n (%)		
	Current drinker	456(42.5)
	Former drinker	73(6.8)
	Nondrinker	545(50.7)

MZ, monozygotic twins; BMI, body mass index; WC, waist circumference; WHR, waist to hip ratio

### Genetic and environmental contributions underlying the associations between dnam and obesity-related measures

Firstly, we conducted bivariate SEMs to estimate the relative contributions of genetic factors to the association between obesity measures and DNAm in the genome-wide scale. Generally, the ACE model exhibited mean AIC value of 3921.97, 5671.81, and − 3411.14 for BMI, WC, and WHR, and was finally applied to 21,701 (5.73%), 60,110 (15.87%), and 186,292 (49.54%) CpG sites, respectively. In contrast, the ADE model demonstrated average AIC values of 3918.65, 5670.60, and − 3488.48, for BMI, WC, and WHR, and was fitted to 356,953 (94.27%), 318,541 (84.13%), and 189,774 (50.46%) CpG sites for these traits.

The average absolute value of the phenotypic correlations across the entire genome was 0.04 for all three obesity measures. Specifically, 176,151, 175,620, and 179,443 CpG sites exhibited negative correlations with BMI, WC, and WHR, respectively. The mean absolute value of the Ra across the genome was 0.09 for BMI, 0.08 for WC, and 0.09 for WHR. Notably, Ra was found to be significant for 30,690, 17,967, and 27,014 CpG sites for BMI, WC, and WHR, respectively. Among these, 14,105 CpGs were correlated with more than one trait, while 3,050 CpGs were correlated with all three traits. Additionally, across the genome, 8,882, 4,901, and 9,635 sites demonstrated significant correlations (Rph > 0.1) with BMI, WC, and WHR, respectively. Particularly, 18 CpGs showed a correlation > = 0.2 with BMI, with the top three CpGs being cg06500161, cg00574958, and cg08309687 (Rph = 0.28, -0.24, and -0.22, respectively). Twelve CpGs had a correlation > = 0.2 with WC, with the highest correlations observed at cg12052203, cg23719534, and cg17612569 (Rph = 0.22, -0.22, and 0.22, respectively). Thirty-nine CpGs exhibited a correlation > = 0.2 with WHR, with the top three CpGs being cg23719534 (Rph = -0.25), cg08035323 (-0.24), and cg26516287 (-0.24) (Supplementary Table 1 [CpGs in Group ①]).

For the CpGs with significant Rph to obesity traits > 0.1, the Ra and Re values derived from bivariate SEMs were illustrated in Fig. 2. As can be seen, the correlations between methylation and obesity metrics for these sites is primarily driven by genetic factors (mean absolute Ra = 0.23, 0.24, and 0.24 for BMI, WC, and WHR, respectively, mean absolute Re = 0.05, 0.03, 0.04), indicating the significant role of genetic influences.

The CpG sites that showed significant Rph values greater than 0.1 and significant Ra values greater than 0.5 for the three obesity phenotypes were presented in Supplementary Tables 2–4 (CpGs in Group ②). These tables include 282 CpG sites correlated with BMI, 241 CpG sites associated with WC, and 402 CpG sites associated with WHR. Among these sites, 89 were associated with more than one obesity measure, and five CpG sites were correlated with all three obesity measures: cg07313626, cg08616667 (*FAM20C*), cg02645905 (*EHMT1*), cg12320621 (*LHPP*), and cg25114752 (*SNORD116-8*). Among CpGs in Group ②, one CpG for WC (cg26516287) exhibited a significant Rph ≥ 0.2 with WC, i.e., overlapped with CpGs in group ①. Two CpGs for WHR (cg26516287 and cg05100634) overlapped with group ①, while no overlap was observed for CpGs with BMI.

### SNP loci corresponding to CpGs and obesity indicators

Additionally, a systematic search of the PubMed and EMBASE databases was conducted to identify relevant GWAS studies that conducted in Asian populations and retrieve the SNP loci that had been previously reported as significantly associated to obesity indicators. The flow chart depicted in Supplementary Fig. 2 followed the Preferred Reporting Items for Systematic Reviews and Meta-Analyses (PRISMA) guidelines and illustrates the literature search process of identifying obesity measures related SNP loci [27]. After inclusion and exclusion, 18 GWAS studies were identified, encompassing 1093, 29, and 302 SNP loci associated with BMI, WC, and WHR, respectively. Detailed information of these studies is provided in Supplementary Table 5. Among these, 604, 9, 175 loci were identified as meQTL in the previous study, corresponding to 2745, 90, 839 CpGs, respectively [23]. Our DNAm dataset encompassed 2,444, 83, and 749 of these loci, which were available for further analyses. Notably, 62 CpGs were found to have a phenotypic correlation > 0.1 with BMI (mean absolute Ra = 0.23), 4 CpGs with WC (mean absolute Ra = 0.17), and 44 CpGs with WHR (mean absolute Ra = 0.23) (Supplementary Table 6 [CpGs in Group ④]).

### Associations between CpG sites and obesity measures and the effects of genetic influences

Firstly, for the CpG sites in group ① that exhibit significant correlations (Rph ≥ 0.2) with obesity metrics, we found that genetic factors play a crucial role in the association between obesity indicators and a certain part of sites (Table 2). Among the 18 CpG sites correlated with BMI, 9 showed significant associations with BMI after FDR correction in the full analysis population, and 6 were significant in the monozygotic twin (MZ) paired analysis. Three of these sites were significant in both analyses: cg06500161 from *ABCG1*, cg25635913 from *HDGF2*, and cg27243685 from *ABCG1*. For WC, 8 out of the 12 correlated CpG sites showed significant associations with WC after FDR correction in the full analysis population, and 1 site was significant in the MZ-paired analysis (cg06500161 from *ABCG1*), which was also significant in the full population. Regarding WHR, 22 out of the 39 correlated CpG sites demonstrated significance in the full population analysis, and 1 site was significant in the MZ-paired analysis (cg23928726 from *PEX10*), which remained significant in both analyses. It is worth stating that for CpGs in group ①, 5 CpGs reported to associated with WHR in the full population were annotated to *PEX10* (cg20664247, cg20823695, cg23626733, cg23629166, and cg23928726).

**Table 2:** CpG sites associated with obesity measures that of phenotypic correlations exceeding 0.2

CPG	CHR	Position	Gene	Locus	Enhancer	dir	Value	*P*	FDR
**BMI**									
**Full Population Analysis**									
cg02100305	11	73,308,991	*FAM168A*	1stExon		+	4.1E-04	1.8E-02	**1.8E-02**
cg04122019	8	21,983,873	*HR*	Body		+	8.7E-04	1.6E-04	**1.8E-04**
cg06500161	21	43,656,587	*ABCG1*	Body	TRUE	+	1.9E-03	2.7E-18	**2.5E-17**
cg09687332	3	49,977,370	*RBM6*	TSS200		+	5.3E-04	3.7E-09	**1.1E-08**
cg14956201	5	14,358,153	*TRIO*	Body		+	1.0E-03	1.9E-05	**3.1E-05**
cg17061862	11	9,590,431	unannotated	unannotated		-	-1.3E-03	2.1E-05	**3.1E-05**
cg25635913	19	4,497,977	*HDGF2*	Body		-	-1.1E-03	7.3E-11	**3.3E-10**
cg27037013	21	35,320,667	unannotated	unannotated	TRUE	-	-1.7E-03	4.1E-05	**5.3E-05**
cg27243685	21	43,642,366	*ABCG1*	5’UTR	TRUE	+	1.3E-03	1.2E-07	**2.7E-07**
**MZ-paired Analysis**									
cg00574958	11	68,607,622	*CPT1A*	5’UTR		-	-5.6E-04	7.6E-03	**1.5E-02**
cg06500161	21	43,656,587	*ABCG1*	Body	TRUE	+	1.3E-03	1.0E-04	**4.1E-04**
cg08309687	21	35,320,596	unannotated	unannotated	TRUE	-	-1.3E-03	2.7E-03	**6.6E-03**
cg11024682	17	17,730,094	*SREBF1*	Body		+	1.5E-03	8.7e-06	**8.5e-05**
cg25635913	19	4,497,977	*HDGF2*	Body		-	-1.4E-03	1.4E-05	**8.5E-05**
cg27243685	21	43,642,366	*ABCG1*	5’UTR	TRUE	+	1.20E-03	4.0E-04	**1.2E-03**
**WC**									
**Full Population Analysis**									
	21	43,656,587	*ABCG1*	Body	TRUE	+	6.1E-04	5.6E-14	**6.2E-13**
cg06710937	13	23,489,940	unannotated	unannotated		-	-3.1E-04	2.5E-02	**3.9E-02**
cg07381872	1	61,408,076	unannotated	unannotated	TRUE	+	2.1E-04	1.2E-02	**3.0E-02**
cg07628841	2	27,851,430	*GPN1;CCDC121*	TSS200;1stExon		-	-2.5E-04	1.5E-02	**3.0E-02**
cg08035323	2	9,843,525	unannotated	unannotated	TRUE	-	-5.5E-04	8.0E-05	**4.4E-04**
cg12052203	11	66,115,045	*B3GNT1*	1stExon		+	3.3E-04	2.8E-02	**3.9E-02**
cg14373579	9	133,455,264	*LOC100272217;FUBP3*	TSS1500;Body		-	-1.3E-04	7.2E-03	**2.6E-02**
cg23719534	15	101,099,284	unannotated	unannotated		-	-2.2E-04	1.6E-02	**3.0E-02**
**MZ-paired Analysis**									
cg06500161	21	43,656,587	*ABCG1*	Body	TRUE	+	4.9E-04	3.4E-04	**4.0E-03**
**WHR**									
**Full Population Analysis**									
cg00695391	1	2,525,548	*MMEL1*	Body	TRUE	-	-2.6E-02	2.9E-02	4.5E-02
cg01787285	1	2,162,682	*SKI*	Body		-	-4.4E-02	6.5E-05	2.5E-04
cg06226150	20	62,369,956	*SLC2A4RG; LIME1*	TSS1500;Body		-	-4.2E-02	2.3E-04	7.0E-04
cg07547549	20	44,658,225	*SLC12A5*	Body		+	6.8E-02	1.9E-08	6.4E-07
cg07628841	2	27,851,430	*GPN1;CCDC121*	TSS200;1stExon		-	-4.3E-02	6.0E-03	1.0E-02
cg08035323	2	9,843,525	unannotated	unannotated	TRUE	-	-1.0E-01	3.3E-06	1.6E-05
cg09469355	1	2,161,886	*SKI*	Body		-	-4.8E-02	1.7E-04	5.7E-04
cg10986043	17	37,820,495	*TCAP*	TSS1500	TRUE	-	-4.0E-02	1.4E-03	3.2E-03
cg11649376	12	81,473,234	*ACSS3*	Body	TRUE	-	-4.2E-02	8.1E-03	1.3E-02
cg13135455	2	241,860,318	unannotated	unannotated	TRUE	-	-6.9E-02	9.2E-07	5.2E-06
cg16395997	1	3,562,798	*WDR8*	Body		+	9.2E-02	4.5E-07	3.0E-06
cg16410171	14	21,511,112	*RNASE7*	5’UTR		-	-4.3E-02	4.3E-05	1.8E-04
cg16932827	3	193,988,639	unannotated	unannotated	TRUE	-	-6.0E-02	1.4E-03	3.2E-03
cg17453456	1	156,094,519	*LMNA*	Body		-	-7.2E-02	4.1E-07	3.0E-06
cg18933331	1	110,186,418	unannotated	unannotated	TRUE	-	-4.8E-02	2.7E-03	5.5E-03
cg20664247	1	2,345,475	*PEX10*	TSS1500		-	-4.9E-02	2.0E-03	4.2E-03
cg20822990	1	17,338,766	*ATP13A2*	TSS1500	TRUE	-	-4.4E-02	1.3E-07	1.4E-06
cg20823695	1	2,345,410	*PEX10*	TSS1500		-	-4.4E-02	1.1E-03	2.9E-03
cg23626733	1	2,345,398	*PEX10*	TSS1500		-	-5.6E-02	3.3E-03	6.3E-03
cg23629166	1	2,345,368	*PEX10*	TSS1500		-	-3.7E-02	5.4E-03	9.7E-03
cg23842572	17	17,030,253	*MPRIP*	Body	TRUE	+	6.8E-02	8.3E-08	1.4E-06
cg23928726	1	2,344,998	*PEX10*	TSS1500		-	-4.3E-02	9.6E-04	2.7E-03
**MZ-paired Analysis**									
cg23928726	1	2,344,998	*PEX10*	TSS1500		+	5.0E-02	1.1E-03	4.4E-02

* Unannotated denotes that the CpG site lacks an associated UCSC reference gene within the Illumina annotation file; + indicates a positive correlation; − indicates a negative correlation

P and FDR denote the *P*-values and *P*-values adjusted for the false discovery rate (FDR)

For the CpG sites from group ②, which exhibit significant Rph > 0.1 and significant Ra > 0.5 with obesity indicators, we found that genetic factors play a primary driving role in the association between obesity metrics and all these sites. In the 282 CpGs correlated with BMI, 155 sites showed significant associations with BMI after FDR correction in the full analysis population. In the MZ-paired analysis, although 23 sites had *p*-values < 0.05, none remained statistically significant after FDR correction (Table 3, Supplementary Table 7 [CpGs in Group ②]). For the 241 CpG sites related to WC, 155 sites were significantly linked to WC after FDR correction in the full analysis population, including 14 sites that were also associated with BMI. In the paired analysis, only one site (cg07136108 from *C11orf9*) remained statistically significant after FDR correction, and this site was not significant in the full analysis population (Table 3, Supplementary Table 8 [CpGs in Group ②]). Among the 402 CpG sites correlated with WHR, 189 sites showed significant associations with WHR after correction in the full population, while only one site (cg07136108) remained statistically significant after correction in MZ twins, consistent with the results for WC, and this site was also not significant in the full analysis population (Table 3, Supplementary Table 9 [CpGs in Group ②]). Additionally, in the full population, the CpG site cg25114752, annotated to *SNORD116-8*, and the CpG site cg12320621, annotated to *LHPP*, were significantly associated with all three obesity indicators.

**Table 3:** Top 5 CpG sites associated with obesity measures that of phenotypic correlations exceeding 0.1 and genetic correlations exceeding 0.5

CPG	CHR	Position	Gene	Locus	Enhancer	dir	Value	*P* ^*^	FDR^*^	*P* ^#^	FDR^#^
**BMI**											
cg10510478	3	137,728,810	*CLDN18*	TSS200		-	-1.2E-03	1.8E-06	**7.0E-05**	4.0E-01	8.9E-01
cg10853830	11	67,266,176	*PITPNM1*	Body		-	-9.4E-04	1.2E-07	**2.6E-05**	4.1E-03	3.3E-01
cg11494616	2	98,561,179	*TMEM131*	Body	TRUE	+	1.1E-03	1.5E-06	**7.0E-05**	5.6E-02	5.8E-01
cg23771088	12	121,975,533	*KDM2B*	Body		+	3.9E-04	7.3E-07	**7.0E-05**	8.9E-01	9.9E-01
cg23866381	4	38,132,555	*TBC1D1*	Body	TRUE	-	-1.3E-03	1.6E-06	**7.0E-05**	7.5E-01	9.6E-01
**WC**											
cg00438616	15	76,304,843	*NRG4*	TSS200		+	1.6E-04	7.2E-06	**2.4E-04**	6.4E-01	9.8E-01
cg05540133	16	88,984,237	*CBFA2T3*	5’UTR		-	-8.0E-04	9.9E-16	**1.8E-13**	2.4E-01	8.6E-01
cg06430309	7	2,397,685	*EIF3B*	Body		+	5.4E-04	2.2E-08	**2.0E-06**	3.3E-01	9.2E-01
cg08361356	10	33,842,206	unannotated	unannotated	TRUE	-	-6.5E-04	1.3E-05	**3.4E-04**	1.5E-01	8.6E-01
cg25105522	17	43,355,089	*MAP3K14*	Body	TRUE	-	-6.7E-04	2.9E-06	**1.3E-04**	4.1E-01	9.6E-01
**WHR**											
cg05540133	16	88,984,237	*CBFA2T3*	5’UTR		-	-1.1E-01	3.2E-13	**9.3E-11**	1.7E-01	8.3E-01
cg06430309	7	2,397,685	*EIF3B*	Body		+	7.3E-02	9.5E-07	**5.5E-05**	5.6E-01	9.9E-01
cg11287987	17	41,837,048	*SOST*	TSS1500	TRUE	-	-5.0E-02	3.2E-07	**2.3E-05**	9.4E-01	1.0E + 00
cg17574785	13	20,933,879	unannotated	unannotated	TRUE	-	-9.0E-02	2.2E-07	**2.1E-05**	2.9E-01	8.7E-01
cg20627971	11	33,759,503	*CD59*	TSS1500		-	-8.7E-02	8.4E-10	**1.2E-07**	4.9E-01	9.9E-01

* Unannotated denotes that the CpG site lacks an associated UCSC reference gene within the Illumina annotation file; + indicates a positive correlation; − indicates a negative correlation

P^*^ and FDR^*^ denote the *P*-values and *P*-values adjusted for the false discovery rate (FDR) in the full population analyses, while P^#^ and FDR^#^ denote the *P*-values and FDR-adjusted *P*-values in the MZ twin-paired analyses

Next, for the CpG sites from group ③, which exhibit significant Rph > 0.1 and nonsignificant Ra < 0.1 with obesity indicators, genetic effects do not play a role or may have a confounding role on the association between the CpG site and the obesity measure. A total of 854, 98, and 553 CpGs were selected for the association analysis with BMI, WC, and WHR, respectively. The results from the bivariate SEMs for these CpGs were detailed on the Supplementary Tables 10–12 (CpGs in Group ③). Among the CpGs in Group ③, three CpGs that phenotypically correlated with BMI (cg01681525, cg06638463, and cg11024682) were also present in Group ①, demonstrating significant Rph ≥ 0.2 with BMI. Additionally, 17 CpGs overlapped with those in Group ①, among which cg23256579, cg23928726, cg20823695, cg27615582, and cg18933331 exhibited the top five highest Rph values with WHR. However, no overlap was identified for WC in this classification.

In the association analysis of CpGs within group ③, for the 854 CpG sites correlated with BMI, 92 sites showed significant associations after FDR correction in the full analysis population, while 127 sites were statistically significant in the MZ-paired analysis, with 89 sites being significant in both (Table 4, Supplementary Table 13 [CpGs in Group ③]). In the 98 CpG sites related to WC, 14 sites were significant in the full analysis population, 6 sites remained significant in the paired analysis, and 4 sites were significant in both (Table 4, Supplementary Table 14 [CpGs in Group ③]). For the 553 CpG sites related to WHR, 134 and 29 sites were significant in the full population and paired population, respectively, including 17 sites significant in both (Table 4, Supplementary Table 15 [CpGs in Group ③]). Among these, cg01945624 and cg16828491, which exhibited associations with WHR in the full-population analysis, as well as cg01945624, which was associated with WC in the full-population analysis, were annotated to the *SH3TC1*.

**Table 4:** Top 5 CpG sites associated with obesity measures that of phenotypic correlations exceeding 0.1 and genetic correlations less than 0.1

CPG	CHR	Position	Gene	Locus	Enhancer	dir	Value	*P* ^*^	FDR^*^	*P* ^#^	FDR^#^
**BMI**											
cg03505562	16	2,077,447	*SLC9A3R2*	Body		+	1.6E-03	1.3E-20	**2.6E-19**	3.7E-05	**6.3E-05**
cg05874450	6	105,627,246	*POPDC3*	5’UTR		+	1.6E-03	9.0E-23	**2.7E-21**	1.7E-03	**2.0E-03**
cg06594281	17	73,083,692	*SLC16A5*	TSS1500		+	1.3E-03	9.4E-23	**2.7E-21**	4.8E-07	**1.6E-06**
cg10324902	3	48,701,666	*CELSR3*	TSS1500		+	1.3E-03	1.0E-22	**2.7E-21**	1.5E-03	**1.8E-03**
cg19615721	14	32,671,389	unannotated	unannotated		+	1.3E-03	4.0E-23	**2.7E-21**	4.7E-05	**7.6E-05**
**WC**											
cg00465247	13	50,703,477	unannotated	unannotated		-	-5.7E-04	2.6E-05	**3.9E-04**	2.0E-01	4.1E-01
cg02837456	11	94,500,612	*AMOTL1*	TSS1500		-	-4.2E-04	6.6E-05	**6.4E-04**	1.6E-03	**1.7E-02**
cg03429645	3	100,053,188	*NIT2*	TSS1500		-	-2.9E-04	3.1E-04	**2.1E-03**	8.5E-01	9.8E-01
cg22858500	10	18,629,006	*CACNB2*	TSS1500		-	-5.2E-04	3.0E-05	**3.9E-04**	9.7E-01	9.9E-01
cg23218957	14	59,109,856	*DACT1*	Body		-	-4.9E-04	1.1E-06	**4.4E-05**	9.6E-01	9.9E-01
**WHR**											
cg00062437	22	23,728,539	unannotated	unannotated		-	-4.2E-02	1.5E-06	**5.7E-05**	3.8E-01	5.4E-01
cg02071600	5	78,808,852	*HOMER1*	1stExon		+	7.0E-02	7.3E-07	**3.4E-05**	2.2E-01	3.9E-01
cg03032770	16	57,669,519	*GPR56*	5’UTR	TRUE	-	-5.4E-02	2.5E-06	**7.6E-05**	6.5E-02	2.0E-01
cg03725309	1	109,757,585	*SARS*	Body		-	-5.1E-02	4.3E-09	**7.9E-07**	6.2E-01	7.6E-01
cg07547549	20	44,658,225	*SLC12A5*	Body		+	6.8E-02	2.4E-08	**2.2E-06**	9.1E-01	9.5E-01

* Unannotated denotes that the CpG site lacks an associated UCSC reference gene within the Illumina annotation file; + indicates a positive correlation; − indicates a negative correlation

P^*^ and FDR^*^ denote the *P*-values and *P*-values adjusted for the false discovery rate (FDR) in the full population analyses, while P^#^ and FDR^#^ denote the *P*-values and FDR-adjusted *P*-values in the MZ twin-paired analyses

Lastly, for the CpG sites in group ④, which present significant Rph > 0.1 and share common SNPs with obesity-related indicators, genetic factors also play a primary driving role in the association between these sites and obesity metrics, similar to the CpG sites in group ①. For the 62 CpG sites that having the common associated SNPs with BMI, 29 sites showed significant associations after FDR correction in the full analysis population, while only 3 sites remained statistically significant in the MZ-paired analysis (Supplementary 16 [CpGs in Group ④]). Conversely, none of the 4 CpG sites related to WC were significant in either the full analysis population or the MZ paired analysis. For the 44 CpG sites shared common SNPs with WHR, 20 sites demonstrated significant associations with WHR after FDR correction in the full analysis population, but only 1 site remained statistically significant in the paired analysis (Supplementary 17 [CpGs in Group ④]).

Subsequently, enrichment analyses were conducted based on CpG groups ①-④. For the CpGs in group ①, 36 significant pathways were identified, 15 of which were obesity-related. These included regulation of lipid storage, positive regulation of cholesterol biosynthetic process, positive regulation of sterol biosynthetic process, lipid storage, positive regulation of cholesterol metabolic process, positive regulation of lipid metabolic process, positive regulation of steroid biosynthetic process, regulation of cholesterol biosynthetic process, regulation of sterol biosynthetic process, cellular response to fatty acid, regulation of cholesterol metabolic process, intracellular lipid transport, response to fatty acid, cholesterol biosynthetic process, and sterol biosynthetic process. Nevertheless, all these obesity-associated pathways were annotated from only 3 genes, namely *ABCG1*, *CPT1A*, and *SREBF1* (Supplementary Table 18).

For the CpGs in groups ②-④, no significant pathways were detected. In these groups, separate enrichment analyses were also attempted for the CpG sites obtained from the full-population and MZ twins respectively. Similarly, no significant pathways were discovered.

## Discussion

In the present study, we report two parts of primary findings regarding the influence of genetic factors on the associations between obesity measures and DNAm. Firstly, the results of genome-wide bivariate SEMs indicate that genetic influences performed a primarily driven effect on the correlations between obesity measures and DNAm, particularly for CpG sites with phenotypic correlations greater than 0.1 with obesity measures. Secondly, by comparing the association analysis results between the full population and the MZ twin-paired population, we found that for most CpG sites with high Ra values in relation to obesity measures, their associations with obesity measures are primarily attributable to genetic factors. In contrast, for CpG sites with lower Ra values, genetic factors either do not contribute significantly to the associations or act primarily as confounders. Additionally, for CpG sites that share common SNPs associated with obesity metrics, similar to those with high Ra values, their associations with obesity measures are also largely influenced by genetic factors.

### Genetic influences predominantly drive the correlations between DNAm and obesity measures

Methylation can heritably change gene expression without altering the DNA sequence and leads to the development and progression of obesity [28]. One study found that in obese individuals, the methylation levels of the *S100A8* and *S100A9* genes exhibit a negative correlation with their expression levels, which is closely associated with the pathological mechanisms underlying obesity [29]. Despite the identification of numerous methylation-associated SNPs, the number of discovered meQTLs remains limited, which may not adequately represent the genetic influence on methylation [30]. In such a context, twin study designs offer unique advantages and have been widely used to investigate the genetic impact on association studies, providing comprehensive genome-wide analysis of the genetic influence on methylation-phenotype associations. To our knowledge, no genome-wide twin studies have been conducted to explore the genetic and environmental correlations between DNA methylation and obesity indicators.

Although obesity is a well-known exposure with a strong and reproducible impact on DNA methylation, the results of this study indicate a mean phenotypic correlation of 0.04 and a genetic correlation of 0.08–0.09 between methylation and obesity at the genome-wide level. Additionally, among sites with phenotypic correlations greater than 0.1, the average genetic correlation was approximately 0.23–0.24, while the environmental correlation was about 0.03–0.05, highlighting the important role of genetic factors in the phenotypic correlation between methylation and obesity. Similar to our findings, a study based on whole blood samples from a twin populations at age 18 established univariate SEMs for 176 CpG sites associated with BMI and found that these sites exhibit high heritability than average (31.4% vs. 23.0%) [31]. Previous research has established that parental BMI can influence offspring DNA methylation patterns, which subsequently impact the offspring’s BMI [32, 33]. Specifically, through blood assessments conducted on offspring, these studies identified that parental BMI influences the DNAm levels at specific CpG sites in umbilical cord blood at birth. Notably, these observed epigenetic modifications were found to persist at 3 and 7 years of age, ultimately shaping long-term BMI trajectories. This finding underscores the significant and lasting role of genetic factors in the relationship between methylation and obesity. Furthermore, numerous studies have identified the epigenetic modifications on various robust genes like the *FTO*, *NBPF3*, and *SREBF1* were related to obesity, suggesting the regulatory role of genetic factors in obesity-associated methylation changes [4, 34]. On the other hand, the epigenetic and genetic alterations involved in the development of obesity are complex. Obesity can induce changes in DNAm, which in turn may cause irreversible perturbations in gene regulation, ultimately affecting subsequent metabolic phenotypes [35, 36]. Therefore, identifying methylation sites that are independent of genetic factors may help elucidate the epigenetic and genetic mechanisms underlying obesity and facilitate the discovery of obesity-related biomarkers with high external validity.

### Genetic influences on the associations between DNAm and obesity measures

The results of this study indicate that, in the absence of controlling for genetic factors, a batch of significantly CpGs that correlated with obesity measures may not represent direct associations. This may also be one of the reasons for the poor extrapolation of obesity-related DNAm markers. From an alternative perspective, several obesity-related CpG sites that have been consistently reported across multiple studies, indicating better reproducibility, such as cg00574958, cg06500161, and cg11024682, exhibit significant associations in MZ-paired analyses [4, 37–39]. Specifically, the cg00574958 and cg11024682 did not show significance in full population analysis without controlling for genetic factors but exhibit significant associations in MZ-paired analyses, suggesting that genetic factors may confound the relationship between obesity and DNAm.

A number of CpGs that associated with obesity while independent of genetic influences were revealed in this study. As mentioned above, cg06500161 was significantly associated with obesity-metrics in both full population and MZ-paired analyses. The cg06500161 was annotated to ATP-binding cassette sub-family G member 1 (*ABCG1*), which facilitates cholesterol and phospholipid efflux from macrophages into high-density lipoprotein (HDL) particles [40]. Previous studies have shown that cg06500161 may mediate the impact of BMI on HDL, confirming that obesity’s influence on related metabolic disorders may act through methylation levels [41]. Cg00574958, associated with carnitine palmitoyltransferase 1 A (*CPT1A*), is linked to triglyceride and blood glycemic levels. Methylation at this site regulates CPT1A expression, playing a crucial role in maintaining blood glucose and lipid metabolism [42–44]. Cg11024682 was annotated to sterol regulatory element binding transcription factor 1 (*SREBF1*), which encodes a transcription factor involved in sterol synthesis and lipid metabolism [45]. Research has indicated that methylation at cg11024682 in *SREBF1* may mediate the association between BMI and non-small cell lung cancer risk [46]. Furthermore, cg08309687 and cg27243685, which have been reported to be associated with BMI in MZ paired analysis (with cg27243685 also demonstrating significance in the full-population analysis), have been identified as being associated with multiple metabolic traits, including liver fat, HbA1c, and lipid profiles, suggesting their linkage to obesity as well [47–49].

By controlling the Ra between obesity metrics and DNAm to be below 0.1, we identified a set of CpG sites that showed significance in both full population and MZ-pair analyses, including a substantial number of novel sites. Many of these CpG sites are located in important regions of their annotated genes, such as TSS200, TSS1500, first exon regions, and enhancer regions. The expression of a proportion of these genes was known to be influenced by methylation levels. For example, cg01074584 is annotated to the TSS1500 region of *SFRP1*, where high methylation in the promoter region is reported to be associated with reduced gene expression [50]. *SREBF1* has been demonstrated to play a pivotal role in the development of obesity and metabolic syndrome. In a study focusing on Chinese children, distinct genetic variants of *SREBF1* exhibited differential responses to cholesterol, thereby contributing to varying risks of obesity and obesity-related metabolic traits [51]. Additionally, *SREBF1* interacts with other genes and metabolic pathways, such as promoting adipogenesis through the regulation of the *VDR/SREBF1* axis, consequently influencing the progression of obesity [52]. Similarly, cg05439191 is annotated to the TSS200 region of the *OGG1* gene, whose expression is also affected by methylation and plays a significant role in obesity-related metabolic pathways [53]. The expression of *DPP4*, annotated by cg09601770 in the first exon region, is influenced by methylation and has been shown to increase in obese individuals, contributing to insulin resistance [54, 55]. Furthermore, several CpG sites are annotated to genes whose expression is influenced by methylation levels and which are associated with other chronic diseases. For example, cg10735632 is annotated to the TSS1500 region of the *C2orf40* gene, which may influence cancer development by downregulating the expression levels of this gene, as reduced expression of *C2orf40* has been implicated in the progression of prostate cancer, breast cancer, lung cancer, and colorectal cancer, where it is associated with tumorigenesis and metastasis [56]. cg05303901 is annotated to the TSS1500 region of the *CREB5* gene, which plays a role in regulating the viral replication [57]. Cg12607106 is annotated to the enhancer region of the *SMYD3* gene, which is involved in lipid metabolism and inhibiting proliferation in cancer cells [58, 59].

Our findings underscored the importance of controlling for genetic influences in the discovery of DNAm-related biomarkers and suggested that future DNAm-related research should carefully consider the influence of genetic factors when establishing and identifying DNAm biomarkers.

### Strengths and limitations

This study has several strengths. First, to the best of our knowledge, it is the first to assess the genetic and environmental correlations between obesity measures and genome-wide DNAm levels. Twin populations serve as a valuable tool for analyzing these correlations. Second, we conducted a comprehensive evaluation of the impact of genetic factors on the relationship between obesity measures and DNAm. By utilizing the twin study design, which allows for the control of genetic factors, we compared the results obtained from association studies in the entire analysis population with those from MZ-paired association studies. This approach enabled us to further investigate the role of genetics in DNA methylation and obesity phenotypes, providing additional control for genetic effects. Third, we systematically searched the PubMed and EMBASE databases to identify SNPs shared by CpG sites and obesity indices, thereby exploring the relationship between genetics, DNAm, and obesity phenotypes. To the best of our knowledge, this is the first study to establish and identify the relationship between CpG sites and obesity phenotypes by identifying common SNP loci.

However, this study has some limitations. First, this study can only provide information on the extent of the genetic effects between DNAm and obesity indices. The specific mechanisms of these effects remain unclear and require further investigation. Second, while this study controlled for genetic factors by adjusting for twin ID to account for relatedness, this approach does not fully address the inherent genetic similarity differences between MZ and DZ twins. This limitation may influence the precision of genetic effect estimates. Third, this study was conducted in a Chinese twin population. Due to ethnic differences and differences in study design, some associations in this study may not be significant. Besides, given the current lack of meQTL studies specifically focused on the Chinese population, we were constrained to utilize data from a study encompassing both European and Asian cohorts to provide meQTL information. This approach may introduce certain biases into the analysis results of CpG sites that identified to have common associated SNPs with obesity measures. Future research should further explore the meQTLs in the Chinese population to obtain methylation quantitative trait locus data. Differences in ancestry are associated with obesity-related characteristics and DNAm, therefore, further research is needed in other populations to elucidate the significance of these CpG sites [60].

## Conclusions

In this study, we conducted a comprehensive examination of the genetic and environmental correlations between genome-wide DNAm and obesity indices. Our findings indicate that the phenotypic, genetic, and environmental correlations between these factors were generally small, with mean values of Rph, Ra, and Re being 0.04, 0.08–0.09, and 0.02–0.03, respectively. However, for CpGs with higher phenotypic correlations (Rph > 0.1), the mean genetic correlations (Ra) were 0.23–0.24, while the mean environmental correlations (Re) were 0.03–0.05, suggesting that the association between DNAm and obesity is primarily driven by genetic factors. To further investigate the associations between DNAm and obesity measures, different strategies were employed, conducting association analyses in both the full population and MZ pairs, where the genetic background was controlled. Firstly, at CpGs with Rph ≥ 0.2, genetic factors were found to drive the association for a subset of these loci. A total of 9, 8, and 22 CpGs were associated with BMI, WC, and WHR, respectively. After MZ twin-pair analysis, only 6, 1, and 1 CpGs remained significant, including cg00574958, cg06500161, cg08309687, cg11024682, cg25635913, cg27243685, and cg23928726. Secondly, at CpGs with Rph > 0.1 and Ra > 0.5, genetic factors predominantly drove the association with obesity indices for almost all sites. In the MZ-paired analysis, none of the significant loci reported in the full population (155 BMI-associated, 155 WC-associated, and 189 WHR-associated sites) remained significant. At loci with Rph > 0.1 and Ra < 0.1, genetic effects either played no role or could have confounded the association with obesity indices. A considerable number of loci remained significant in both the entire population analysis and the MZ twin-pair analysis. Finally, at CpG sites with Rph > 0.1 and common associated SNPs, genetic factors were similarly found to predominantly drive the association with obesity indices. Out of 29 BMI-related, 0 WC-related, and 20 WHR-related CpGs identified in the full population, only 3 BMI-related and 1 WHR-related CpGs remained significant in the MZ-paired analysis. Our results highlight the significant role of genetic factors in the analyses of the relationship between DNAm and obesity. This conclusion may contribute to the low replicability of obesity-related DNAm markers found in previous studies. Warranted caution is advised in future research for the interpretation of DNAm-related biomarkers.

## Electronic supplementary material

Below is the link to the electronic supplementary material.

Supplementary Material 1

Supplementary Material 2

## Data Availability

The datasets analyzed in this study are not publicly available due to the informed consent involved in this study stating that the data of the participants could not be disclosed to a third party. But the data that support the findings of this study are available on request from the corresponding author Prof. Wenjing Gao.
