# Supplementary material for: Genetic influences on the association between DNA methylation and obesity measures: insights from a twin study design
Source: Cell Biosci. 2025 Jul 23;15:108. doi: 10.1186/s13578-025-01446-2 (PMC12288357; doi:10.1186/s13578-025-01446-2)
Supplement: Supplementary file 2 — Supplementary Material 2 [file 13578_2025_1446_MOESM2_ESM.docx]

**Supplementary Text**

**The analysis process of DNA methylation data**

Methylation levels of each CpG sites were reported as β-values in this study, representing average methylation level ranging from 0 (fully unmethylated) to 1 (fully methylated). β-values were computed using the formula: β= M/(M+U+100), wherein M and U represent respectively for the average probe signal intensity at each site in the methylated and unmethylated states, which were assessed utilizing the R package “minfi”^1^. Subsequently, the β-values were quantile normalized, followed by adjustment for blood cell proportions (CD4T, CD8T, Mono, NK, Bcell, and Gran) through implementation of the (version 2.18.3)^2^. Quality control (QC) measures were implemented for the DNAm data to filter out low-quality detection probes and samples from the DNAm dataset. These included: (1) identification of missing probes that no significant differences were observed (P> 0.01) between the signals from the CpG site and the blank control from probes; (2) exclusion of probes with minor allele frequency (MAF) > 0.01 or those that had annotated single nucleotide polymorphisms (SNPs) on the microarray; (3) removal of missing probes with a detection P> 0.01 in more than 1% of the samples; (4) elimination of cross-reactive probes; (5) deletion of samples with missing rates exceeding 1% in probes.

**Systematically search process**

A systematic review was performed in the PubMed database for genome-wide association studies (GWASs) on obesity measures using the following search terms.: ((chinese[Title/Abstract]) OR (asian[Title/Abstract])) AND ((gwas[Title/Abstract]) OR (genome-wide association study[Title/Abstract])) AND ((Body Mass Index[Title/Abstract]) OR (BMI[Title/Abstract]) OR (waist circumference[Title/Abstract]) OR (waist circumference to hip ratio[Title/Abstract]) OR (Waist-Hip Ratio ratio[Title/Abstract])). The Embase database was searched by the following terms: ('BMI'/exp OR 'Body Mass Index'/exp OR 'waist circumference'/exp OR 'waist circumference to hip ratio'/exp OR 'WHR'/exp OR 'waist to hip ratio'/exp) AND ('gwas'/exp OR 'genome-wide association study'/exp) AND ('Chinese'/exp OR 'Asian'/exp) AND ('article'/it OR 'article in press'/it OR 'preprint'/it OR 'short survey'/it).

The systematic searches were all performed until November 18, 2024. We evaluated the efficiency of each publication's title and abstract in selecting corresponding single nucleotide polymorphisms (SNPs). We identified 254 relevant publications in PubMed (200) and Embase (216). The GWASs on body mass index(BMI), waist circumference (WC), and waist to hip ratio (WHR) were selected and included for the identified associations. Studies focused on irrelevant phenotypes or other types of obesity like maternal obesity were excluded. Studies on non-Asian populations, children, and animals were excluded. Studies with a sample size of less than 500 for GWAS analysis were also excluded. However, studies that performed validation analyses of SNP loci identified in GWAS studies of other populations within Asian populations were included. Mendelian randomization research and bivariate GWASs were excluded. Studies specifically targeting the genetic variants of particular genes or loci, or ultimately identified no significantly associated loci related to obesity measures were excluded. In addition, systematic reviews, commentaries, abstracts, and editorial letters were also excluded from our analysis.

**Methylation quantitative trait loci used in this study**

The SNP loci identified through GWAS searches as related to obesity phenotypes and confirmed as methylation quantitative trait loci (meQTLs), have been included along with their corresponding CpG sites. These data are derived from a published study^4^.

**Supplementary Table 5 Characteristics of GWASs on obesity-related indicators**

| **Reference** | **Population for DNA methylation analysis** | **Female/male** | **Phenotype** | **Covariates included in analysis** | **Significant levels** | **No. of CpGs identified** |
| --- | --- | --- | --- | --- | --- | --- |
| Cho et al, 2009^5^ | 8,842 individuals from Korea | 4659/4183 | BMI | Age, sex and recruitment area | 1.0 × 10^−5^ | 3 |
| As above | As above | As above | WHR | As above | As above | 6 |
| Croteau-Chonka et al, 2011^6^ | 1,895 female participants from the Philippines | 1,895/0 | BMI | Age, actual time in years since baseline study visit, assets, income, urbanicity index, menopause status, months since the previous visit spent lactating or pregnant, current lactation status, and activity level | 5.0 × 10^−8^ | 8 |
| As above | As above | As above | WC | As above | As above | 9 |
| Dorajoo et al, 2012^7^ | 10482 individuals from Chinese, Malay and Indian ethnic groups from Singapore | 6100/4382 | BMI | - | 1.0 × 10^−5^ | 15 |
| Dorjgochoo et al, 2011^8^* | 6922 women from Shanghai | 6922/0 | BMI | Age, square of age, menopausal status and sample set and disease status | 0.05 | 2 |
| Okada et al, 2012^9^ | 26,620 Japanese individuals from the BioBank Japan | 12378/14242 | BMI | Gender, age, age-squared, smoking history, the affection statuses of the diseases and the demographic classifications of the medical institutes | 5.0 × 10^−5^ | 9 |
| Hong et al, 2012^10^* | 1040 individuals living in eastern area of China | 675/365 | BMI | Age, sex | 0.0022 | 23 |
| Kim et al, 2013^11^ | 1,049 individuals from Mongolia | 544/505 | BMI | Age, sex | 0.01 | 6 |
| As above | As above | As above | WC | As above | As above | 5 |
| Yang et al, 2014^12^ | 597 Northern Chinese individuals | 339/258 | BMI | Age, sex | FDR<0.05 | 8 |
| Wen et al, 2014^13^ | 86, 757 individuals of Asian ancestry | 46502/40255 | BMI | Age, sex | 7.59 × 10^−6^ | 8 |
| Wang et al, 2016^14^* | 2958 subjects from Chinese community-based populations | 1606/1352 | BMI | Age, sex | 0.05 | 31 |
| As above | As above | As above | WC | As above | As above | 6 |
| As above | As above | As above | WHR | As above | As above | 13 |
| Akiyama et al, 2017^15^ | 173,430 Japanese individuals from the BioBank Japan | 82,438/90,992 | BMI | Age, sex, smoking (ever or never), top 10 PCs, affected disease | 1.0 × 10^−6^ | 85 |
| Cho et al, 2020^16^ | 10,038 Korean people from the Korean Health and Genome Study | 3,013/7025 | BMI | Age, residential area, smoking, alcohol habit and exercising | 1.0 × 10^−3^ | 3 |
| Liu et al, 2021^17^ | 24,000 individuals from the Taiwan Biobank | - | BMI | - | 5.0 × 10^−8^ | 33 |
| Wong et al, 2022^18^ | 21,978 subjects in Taiwan Biobank | 11022/10,956 | BMI | Age, age^2^, sex, and top 10 PCs | 5.0 × 10^−8^ | 23 |
| As above | As above | As above | WC | As above | As above | 5 |
| Wang et al, 2022^19^ | 65,689 individuals from Taiwan Biobank | - | BMI | Age, sex, age by sex interaction, and top 20 PCs | 5.0 × 10^−8^ | 26 |
| As above | As above | As above | WHR | As above | As above | 20 |
| Kim et al, 2022^20^ | 1,937 Korean individuals screened by Seoul National University Hospital | 1437/480 | BMI | Site of recruitment and age | 1.0 × 10^−5^ | 4 |
| O'Loughlin et al, 2023^21^ | 100,377 Chinese individuals from the China Kadoorie Biobank | 57,443/42,934 | BMI | - | 1.0 × 10^−8^ | 838 |
| As above | As above | As above | WHR | As above | As above | 263 |
| Jo et al, 2024^22^ | 85,947 Korean subjects from four Korean cohorts | 51,317/34,630 | BMI | Age, sex, and the first 10 PCs | 5.0 × 10^−8^ | 15 |
| As above | As above | As above | WHR | As above | As above | 5 |

*Validation analyses of SNP loci identified in GWAS studies of other populations that conducted using Asian populations.

BMI, body mass index; WC, waist circumference; WHR, waist to hip ratio


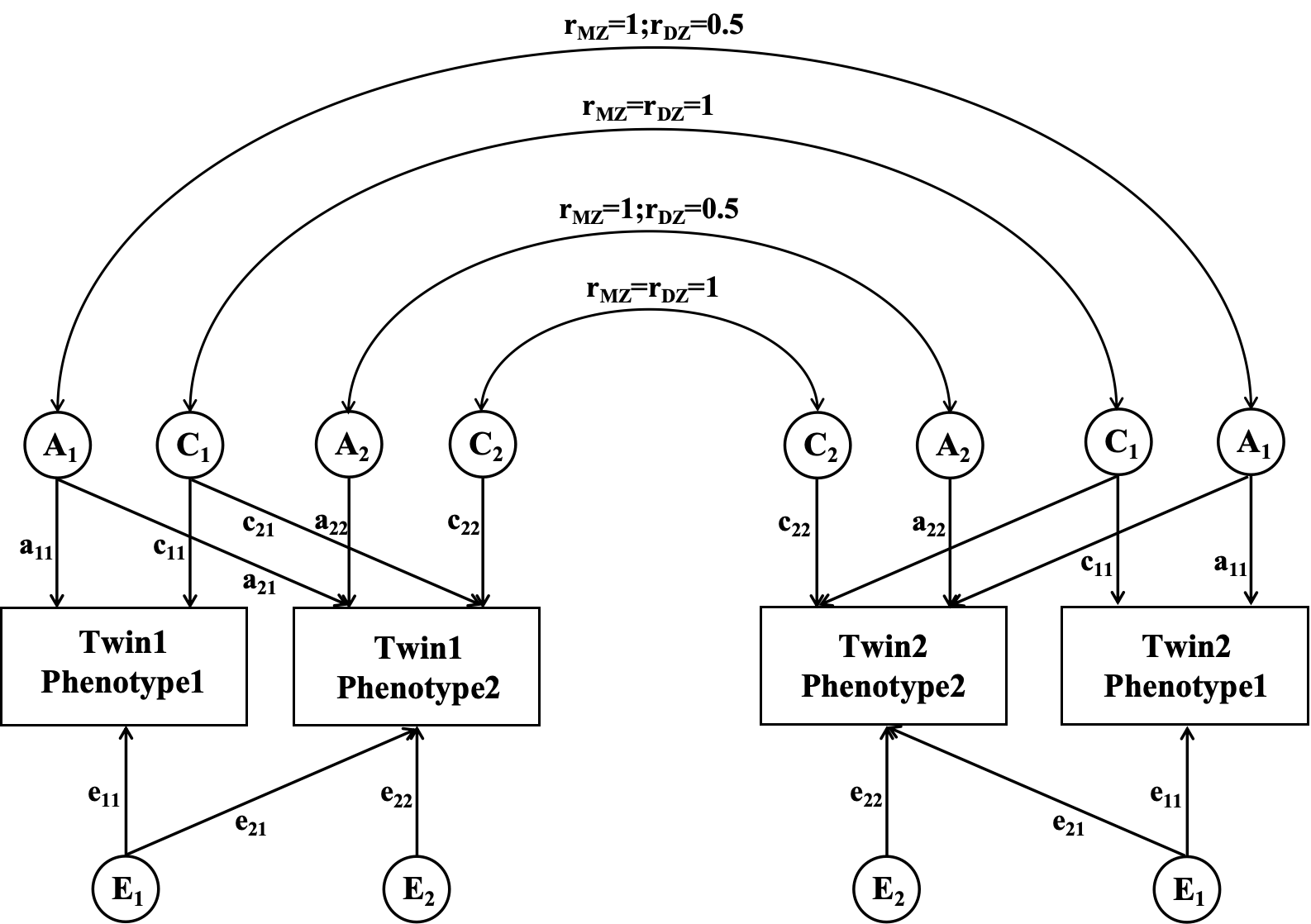


**Supplementary Figure 1. Diagram for the bivariate structural equation model**

The phenotypes for a twin pair are depicted in squares, while latent factors are in circles. The correlations of additive genetic variance (A) are 1 in MZ twins and 0.5 in DZ twins. For both MZ and DZ twins, the correlations of common environmental variance (C) are 1. Unique environmental variance (E) is consistently 0.


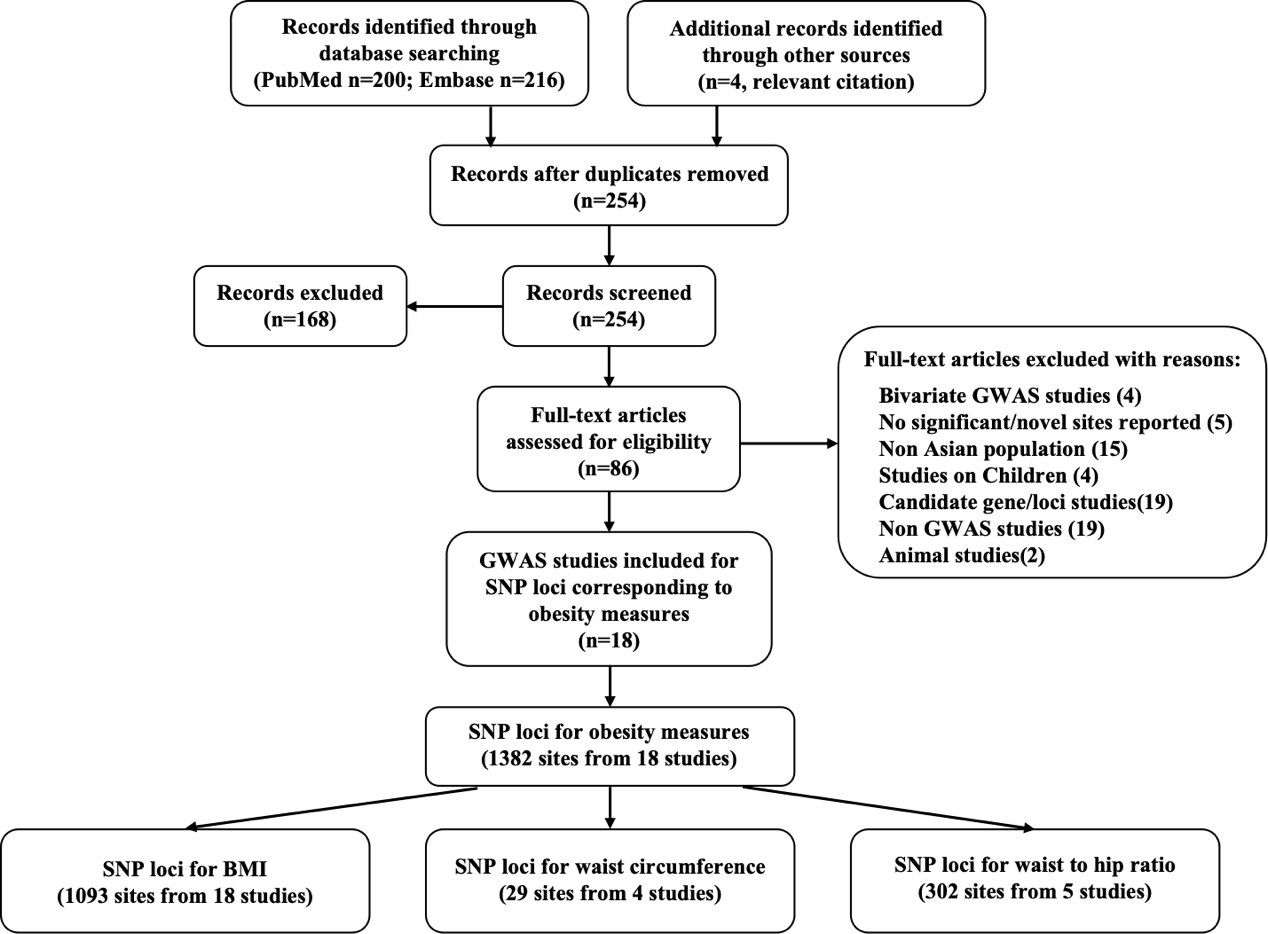


**Supplementary Figure2. PRISMA 2009 flow chart of the study selection process**

SNP indicates single nucleotide polymorphisms; GWAS, genome-wide association study; BMI, body mass index; WC, waist circumference; WHR, waist to hip ratio.

**Reference**

1. Aryee MJ, Jaffe AE, Corrada-Bravo H, et al. Minfi: a flexible and comprehensive Bioconductor package for the analysis of Infinium DNA methylation microarrays. *Bioinformatics.* 2014;30(10):1363-1369.

2. Tian Y, Morris TJ, Webster AP, et al. ChAMP: updated methylation analysis pipeline for Illumina BeadChips. *Bioinformatics.* 2017;33(24):3982-3984.

3. Leek JT, Johnson WE, Parker HS, Jaffe AE, Storey JD. The sva package for removing batch effects and other unwanted variation in high-throughput experiments. *Bioinformatics.* 2012;28(6):882-883.

4. Hawe JS, Wilson R, Schmid KT, et al. Genetic variation influencing DNA methylation provides insights into molecular mechanisms regulating genomic function. *Nat Genet.* 2022;54(1):18-29.

5. Cho YS, Go MJ, Kim YJ, et al. A large-scale genome-wide association study of Asian populations uncovers genetic factors influencing eight quantitative traits. *Nature Genetics.* 2009;41(5):527-534.

6. Croteau‐Chonka DC, Marvelle AF, Lange EM, et al. Genome‐Wide Association Study of Anthropometric Traits and Evidence of Interactions With Age and Study Year in Filipino Women. *Obesity.* 2012;19(5):1019-1027.

7. Dorajoo R, Blakemore AIF, Sim X, et al. Replication of 13 obesity loci among Singaporean Chinese, Malay and Asian-Indian populations. *International Journal of Obesity.* 2011;36(1):159-163.

8. Dorjgochoo T, Shi J, Gao YT, et al. Genetic variants in vitamin D metabolism-related genes and body mass index: analysis of genome-wide scan data of approximately 7000 Chinese women. *International Journal of Obesity.* 2011;36(9):1252-1255.

9. Okada Y, Kubo M, Ohmiya H, et al. Common variants at CDKAL1 and KLF9 are associated with body mass index in east Asian populations. *Nature Genetics.* 2012;44(3):302-306.

10. Hong J, Shi J, Qi L, et al. Genetic susceptibility, birth weight and obesity risk in young Chinese. *International Journal of Obesity.* 2012;37(5):673-677.

11. Kim H-J, Yoo YJ, Ju YS, et al. Combined linkage and association analyses identify a novel locus for obesity near PROX1 in Asians. *Obesity.* 2013;21(11):2405-2412.

12. Yang F, Chen XD, Tan LJ, et al. Genome wide association study: searching for genes underlying body mass index in the Chinese. *Biomed Environ Sci.* 2014;27(5):360-370.

13. Wen W, Zheng W, Okada Y, et al. Meta-analysis of genome-wide association studies in East Asian-ancestry populations identifies four new loci for body mass index. *Hum Mol Genet.* 2014;23(20):5492-5504.

14. Wang T, Ma X, Peng D, et al. Effects of Obesity Related Genetic Variations on Visceral and Subcutaneous Fat Distribution in a Chinese Population. *Scientific Reports.* 2016;6(1).

15. Akiyama M, Okada Y, Kanai M, et al. Genome-wide association study identifies 112 new loci for body mass index in the Japanese population. *Nat Genet.* 2017;49(10):1458-1467.

16. Cho H-W, Jin H-S, Eom Y-B. Association between non-Caucasian-specific ASCC1 gene polymorphism and osteoporosis and obesity in Korean postmenopausal women. *Journal of Bone and Mineral Metabolism.* 2020;38(6):868-877.

17. Liu YS, Wu PE, Chou WC, et al. Body mass index and type 2 diabetes and breast cancer survival: a Mendelian randomization study. *Am J Cancer Res.* 2021;11(8):3921-3934.

18. Wong HS, Tsai SY, Chu HW, et al. Genome-wide association study identifies genetic risk loci for adiposity in a Taiwanese population. *PLoS Genet.* 2022;18(1):e1009952.

19. Wang SH, Su MH, Chen CY, et al. Causality of abdominal obesity on cognition: a trans-ethnic Mendelian randomization study. *Int J Obes (Lond).* 2022;46(8):1487-1492.

20. Kim HJ, Son HY, Sung J, et al. A Genome-Wide Association Study on Abdominal Adiposity-Related Traits in Adult Korean Men. *Obes Facts.* 2022;15(4):590-599.

21. O'Loughlin J, Casanova F, Fairhurst-Hunter Z, et al. Mendelian randomisation study of body composition and depression in people of East Asian ancestry highlights potential setting-specific causality. *BMC Med.* 2023;21(1):37.

22. Jo J, Ha N, Ji Y, et al. Genetic determinants of obesity in Korean populations: exploring genome-wide associations and polygenic risk scores. *Brief Bioinform.* 2024;25(5).
